# Eva Viola Mead

**Published:** 1912-12

**Authors:** 


Eva Viola Mead was born at East Otto, Cattaraugus County, in 1871. In 1884 her family moved to Springville, N. Y., where Eva entered the high school. She was a brilliant pupil, graduating at the age of sixteen. She immediately began teaching, in which work she was successful from the first. She taught for eight or nine years in Cattaraugus Country. In 1896 she came to Buffalo and began teaching at No. 51. She was put in charge of the ninth grade in 1899 and continued in this school until she gave up teaching for the practice of medicine.

Miss Mead entered the University of Buffalo in 1892. Her first year in college was followed by a year or two of teaching, in order to earn the necessary money for her college expenses. After she began to teach in Buffalo she again took up her studies in the University, and graduated in 1900. In 1903 she opened her office at 426 Dearborn Street. She continued to practice medicine on Dearborn Street until last October when she moved to her new home, 241 West Ferry Street.

Her confreres respected her judgment, knew her for a successful physician who ably and conscientiously cared for a large practice. But the thing for which she will longest be remembered was her warm heart. She practiced much among the lowly, knew what life meant to the poor and was ever ready to serve the humblest with every means at her command—skill, time, money. Hers was a great character, simple direct, without guide or pretense, unconscious of her own worth.

